# POLICY SERIES: TRENDS IN ELDER ABUSE AND INITIATIVES TO PREVENT ELDER ABUSE IN JAPAN DURING THE PAST 20 YEARS

**DOI:** 10.1093/geroni/igae098.1847

**Published:** 2024-12-31

**Authors:** Michelle Putnam, Pamela Teaster

**Affiliations:** Chair; Discussant

## Abstract

This symposium begins by briefly introducing activities of Japan Academy for the Prevention of Elder Abuse, cerebrating 20 years, and features the Japanese Act on the Prevention of Elder Abuse. First, using data collected by the Ministry of Health, Labour, and Welfare (MHLW), research analyses present trends in elder abuse case reports substantiated as “elder abuse”, trends in reporters in both institutional and domestic settings, and introduce recent amendments of operational standards made by ministerial ordinance. Second, using data (N=171) on elder abuse from Matsudo-City collected in 2019, analysis examines relationships between trends in the increasing number of elder abuse reports made by the police (about 43% of all cases) and trends in the number of cases regally considered to be “elder abuse” (only 17.8% of those reported to the police were legally considered to be “elder abuse”). It examines possible reasons and propose necessary policy changes in definitions of elder abuse. Third, using longitudinal data collected by Matsudo-city and by the MHLW from 2006 through 2022, this analysis examines the impacts of COVID-19 on the occurrence of elder abuse. Analyses revealed that neither data set showed significant increase in frequencies of occurrence of elder abuse due to COVID-19. Rather, it showed a decrease for the MHLW data. This study examines possible reasons and make recommendations to further decrease frequencies of occurrence of elder abuse cases. Abuse, Neglect and Exploitation of Older Persons Interest Group Sponsored Symposium

